# Growth and structural discrimination of cortical neurons on randomly oriented and vertically aligned dense carbon nanotube networks

**DOI:** 10.3762/bjnano.5.169

**Published:** 2014-09-17

**Authors:** Christoph Nick, Sandeep Yadav, Ravi Joshi, Christiane Thielemann, Jörg J Schneider

**Affiliations:** 1 University of Applied Sciences Aschaffenburg, Department of Engineering, BioMEMS lab, Würzburger Strasse 45, 64743 Aschaffenburg, Germany; 2 Technische Universität Darmstadt, Fachbereich Chemie, Eduard-Zintl-Institut für Anorganische und Physikalische Chemie, Alarich-Weiss-Str. 12, 64287 Darmstadt Germany

**Keywords:** carbon nanotube, chemical vapour deposition, interface, neuron, scaffold

## Abstract

The growth of cortical neurons on three dimensional structures of spatially defined (structured) randomly oriented, as well as on vertically aligned, carbon nanotubes (CNT) is studied. Cortical neurons are attracted towards both types of CNT nano-architectures. For both, neurons form clusters in close vicinity to the CNT structures whereupon the randomly oriented CNTs are more closely colonised than the CNT pillars. Neurons develop communication paths via neurites on both nanoarchitectures. These neuron cells attach preferentially on the CNT sidewalls of the vertically aligned CNT architecture instead than onto the tips of the individual CNT pillars.

## Findings

Biochemically functionalised carbon nanotubes (CNTs) are attractive for various sensing and electronic applications. These include, but are not limited to, gas sensors [1], mechanical sensors [2], biosensors (e.g., for glucose or DNA) [3–4], and vertical interconnect access (vias) applications based on CNT bundles [5]. CNTs have also sparked interest in the biomedical community since they have outstanding potential as a substrate for growing different cell type materials [6–8]. Due to their very good electrical conductivity they are a promising substrate for neuron growth as well as for biocompatible electrode materials to record and/or stimulate neural activity. CNTs do have a high capacity and low impedance, e.g., compared to IrO_2_ which is widely used as electrical interface for cells, as has been manifested by cyclic voltammetry and impedance spectroscopy [9]. Thus CNTs allow to minimise the stimulation voltage as well as the electrode sizes while maintaining a good performance in signal recording [10]. This subject has been of strong interdisciplinary interest over the last decade [9,11–12]. Mainly unordered randomly deposited CNTs and dense carbon nanotube fibres (CNF) have been studied as growth substrate for cells. It was found that both carbon based materials can boost neural activity, most likely due to short circuiting the cells and thus increasing excitatory input [13–15]. Furthermore, due to their 1D morphology, the topography of CNTs may resemble the extracellular matrix, therefore increasing the adhesion of neurons [16]. In addition, chemical functionalization [17] and surface charges [18] influence neurite outgrowth and network formation. CNT islands can serve as scaffolds for neurons due to their well-known capability to guide neural growth [9,19–21]. Since CNTs offer a high intrinsic surface area and thus a high charge injection capability they have proven their ability as electrode material for the recording and the stimulation of neurons. For this purpose randomly oriented CNTs have been synthesized and transferred to suitable substrates involving techniques as stamping/printing and in-place growth. The use of vertically aligned CNTs for the design of 3D electrodes was proposed [22]. The interface between dense vertically aligned carbon nanofibers and neurons derived from the rat pheochromocytoma (PC12 cell line) was also studied [10]. However, although vertically grown CNTs have been proposed as stimulating electrodes for neurons, the growth of primary neurons on these nanostructures has not yet been studied in depth and may present a substantial step along these lines. Herein, we report on the in vitro growth of cortical neurons on islands of randomly oriented CNT as well as on islands of vertically aligned up to 500 µm in length CNT structures. For the fabrication of both types of CNTs a water-assisted catalytically driven chemical vapour deposition (CVD) growth process was employed [23]. Two different morphologies of CNT arrays were grown depending on the CVD conditions applied (i.e., Si and Au substrate, metal catalyst, gas volume). Subsequently both types of spatial arrays, randomly oriented and ordered vertically aligned 3D CNT architectures were studied towards their interaction towards cultured neurons (general growth behaviour, preferential growth).

**Figure 1:**
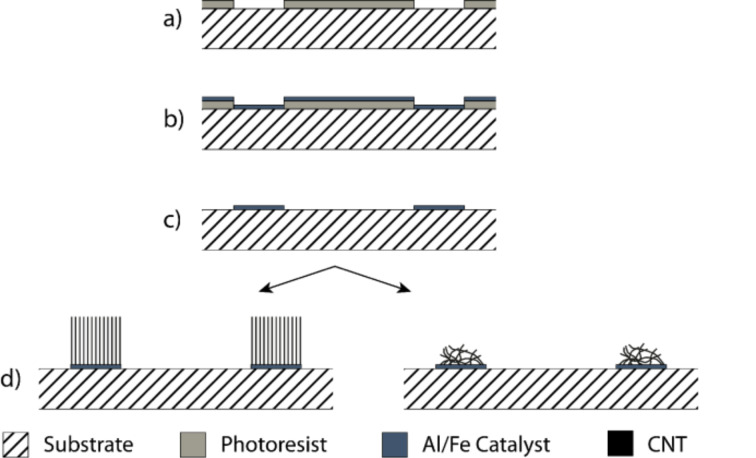
General scheme for the fabrication of spatially deposited CNT islands. (a) A photoresist is lithographically structured; b) defined aluminium and iron layers were evaporated onto the substrate. After removing the photoresist the catalyst islands of bimetallic Al/Fe are spatially structured on the silicon substrate. (d) The follow up growth of CNTs proceeds selectively on these spatially defined catalyst islands in a vertically aligned fashion on the Si substrate (left side), or in a randomly oriented fashion on an Au substrate (right side).

Islands of randomly oriented and vertical aligned CNTs were structured by using a photo-lithography process as depicted in Figure 1 [24]. A photosensitive resist (AZ 701 mir, MicroChemicals GmbH, Ulm, Germany) was spin-coated onto a respective substrate and structured by exposure through a mask and subsequent development. A thin film bimetallic catalyst layer (10–12 nm Al and 1.0–1.4 nm Fe) was first deposited by using electron beam evaporation onto the substrate following by a removal of the resist. This left spatially deposited islands of catalyst behind. Catalyst annealing and CNT synthesis was subsequently carried out in a quartz tube furnace at atmospheric pressure. Typical growth conditions are 780 °C under an argon/hydrogen/ethene/water atmosphere for 2–3 minutes. After heating the substrates to the growth temperature under a steady flow of hydrogen (400 sccm), argon (600 sccm), and ethene (75 sccm) together with a controlled amount of water vapour was introduced into the CVD reactor by streaming a small amount of carrier gas through a water bubbler (150–200 ppm by dew point sensor measurement). Randomly oriented CNT islands were synthesised on silicon substrates covered with a thin (100 nm) layer of gold. This assures a random CNT growth. In contrast, for the growth of the aligned CNT arrays, the growth substrate was silicon and the growth temperature was set to 800 °C with an ethylene flow of 100 sccm (all other conditions being the same as above). The CNTs obtained in this process are mainly double-walled as characterized and described earlier by us [23].

Neural cell culturing was performed by using cryo-conserved embryonic cortical rat neurons (E18 and E19) (Lonza Ltd, Basel, Switzerland). The substrates with CNT islands were sterilized under UV-light [25]. Cell culture medium was prepared according to the following protocol: L-glutamine (2 mM) and 2% NSF-1 were added to the PNBM basal medium. NSF-1, a supplement supporting neuronal growth and survival, was aliquoted, frozen and added to the medium immediately before each use. Neurons were thawed and cultured with a density of approximately 500,000 cells/mm^2^ onto each substrate and incubated in a humidified atmosphere for 4 h. Finally, petri dishes were filled with approximately 3 mL culture medium. Half the medium was replaced twice a week and completely replaced every second week.

**Figure 2:**
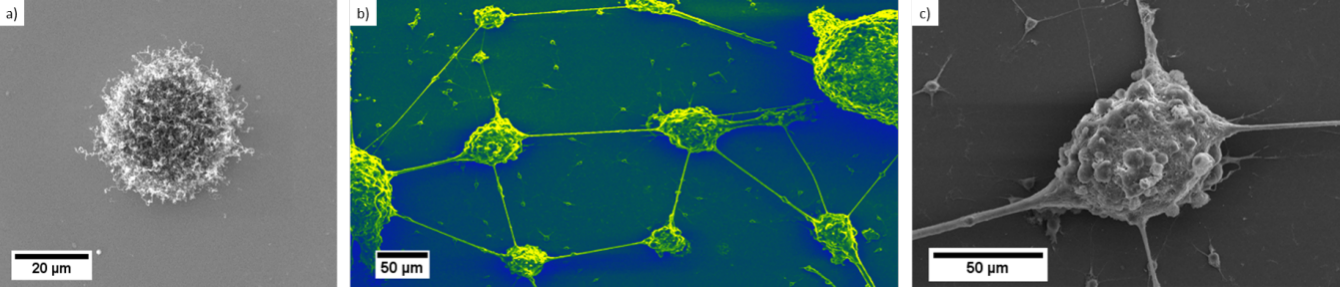
SEM images of neurons cultured on randomly oriented CNT islands. Panel a) depicts a single CNT island with a diameter of ca. 30 µm. In b) and c) after 21 days neurons have accumulated in vitro onto those CNT islands. Neuron cells prefer to grow and adhere onto the CNT islands, forming intimate clusters around individual CNT islands with strong interconnections between the islands (the distance from CNT centre to CNT centre is 200 µm).

In the cell culture protocol no coating (e.g., laminin or poly-D-lysine) prior to cell cultivation was performed. The cell growth behaviour in vitro was studied by scanning electron microscopy (SEM) on pristine vertically aligned CNT arrays and compared to growth of cortical neurons on islands of randomly oriented CNTs under the same conditions. The cells had to be fixed and finally dried before being sputter coated with 5 nm PtPd for SEM investigation. For doing so, the cell culture medium was removed from the substrates and replaced by 2.5% glutaraldehyde. After 2.5 h glutaraldehyde was removed and all substrates were washed in DI-water three times for 20 min each. After this fixation cells were dried in ethanol (10%, 30%, 50%, 70%, 90%, 99.6% for 20 min each). Finally, ethanol was replaced by hexamethyldisilazane (HMDS) that was left to dry in air [26–27]. The obtained samples were subsequently studied by SEM (Philips XL 30 FEG at 15–20 kV). It is obvious that neurons are strongly attracted by the randomly oriented CNT islands and adhere exclusively onto the nanostructured areas (see Figure 2). The cells form dense networks around the islands and also inter-island connections consisting of neurits and cells.

**Figure 3:**
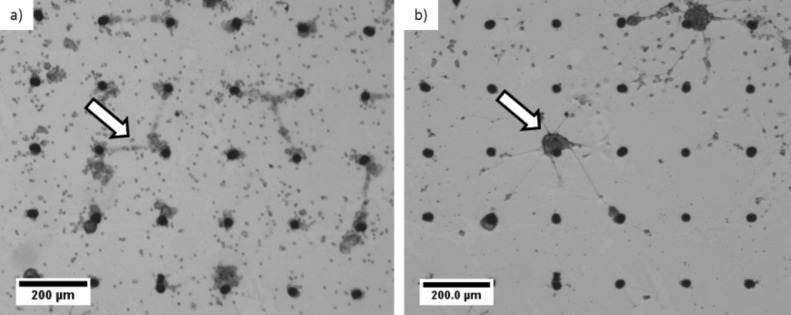
Growth of cortical neurons cultured on islands of vertically aligned CNT architectures. a) Formation of neuron clusters after 4 h and b) after 14 days in vitro. Each CNT pillar is 30 µm in diameter and the distance between two pillars is 200 µm (centre to centre).

Cell motility and network formation of neurons cultured on islands of vertically aligned CNTs were studied for a time period of 14 days after cultivation (Figure 3). After 4 h of cultivation, the neurons already formed dense clusters in the vicinity of individual CNT pillars (Figure 3a). Occasionally connections between cell clusters were formed (Figure 3a, marked by the arrows). Nevertheless, cells in the interspace region between CNT pillars on the silicon substrate are visible, connecting the pillars. After 2 days in vitro, these “cell bridges” had disappeared but originally formed clusters had attracted even more cells and thus appeared larger. Cells that were initially attached to the silicon substrate disappeared and significantly fewer cells are observed in the interspace between the CNT pillars. After 7 days in vitro the majority of the cells in the interspaces, however, have disappeared and dense cell clusters around the CNT were then pronounced.

**Figure 4:**
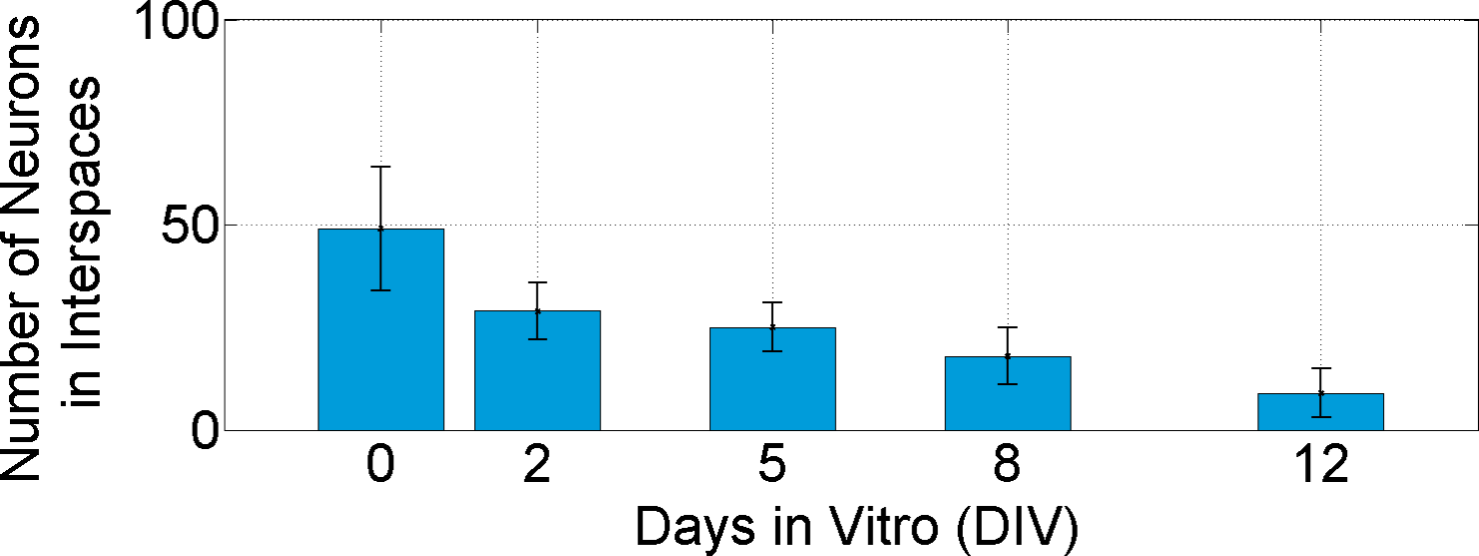
Development of the number of neurons in the interspace regions of the spatially oriented CNTs.

Every second day single non-aggregated cells in more than ten interspaces were counted and averaged. Four hours after seeding 49 ± 15 neural cells could be observed in an interspace area of (200 µm × 200 µm – π·(15 µm)^2^) = 0.393 mm^2^. This number dropped to 9 ± 6 from day 12 onwards. Figure 4 summarizes the development of the number of neurons in the interspaces. At the same time the clusters around the CNT structures appeared darker representing an increased density of cells in the z-axis. This shows that cells grew into three dimensional networks around the CNT islands. Neural connections between CNT pillars could also be observed and became clearly visible after 14 days in vitro (Figure 3b arrow marked).

Neurons accumulate in dense networks around the vertical pillars of CNT and form connections via neurites and in some cases also through some cell bodies between the aggregated networks. These results are in line with the results after using micro-stamped structures of carbon nanotubes, which led to the self-assembly of neural networks [28] and to the formation of neural networks on islands of unordered CNTs [12].

**Figure 5:**
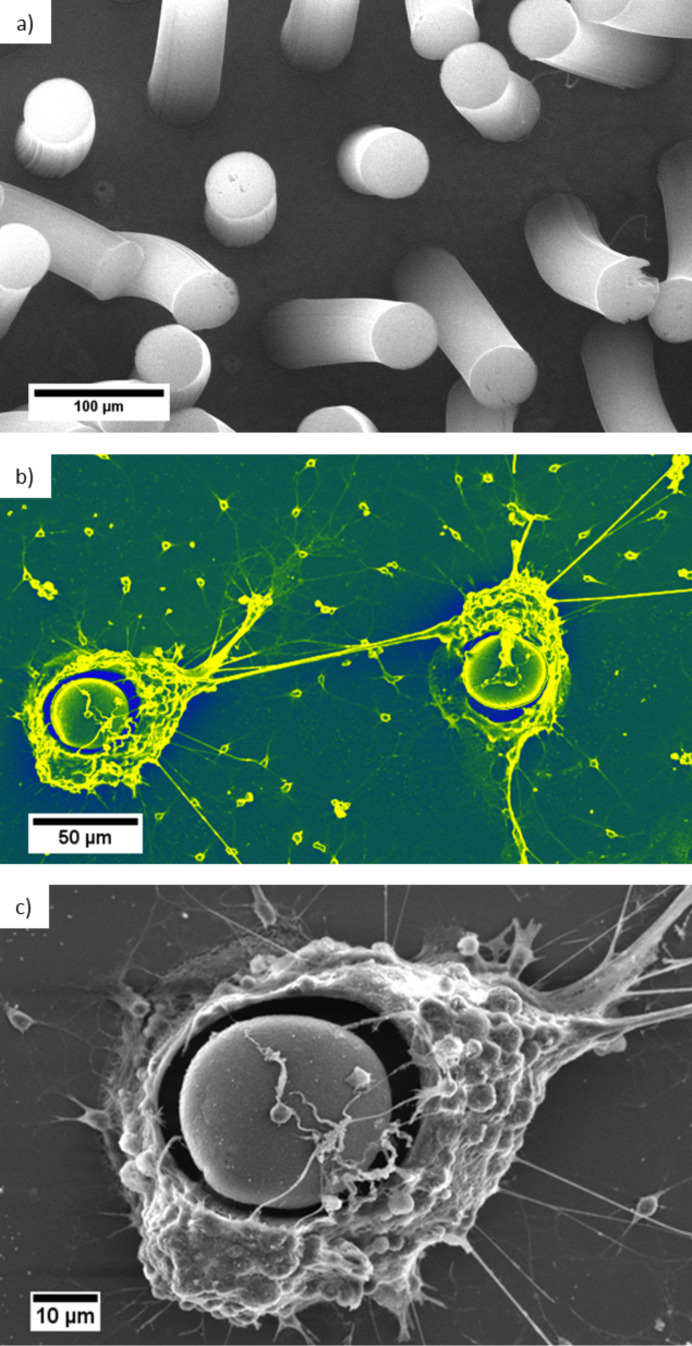
SEM images of a) typical size and arrangement of CNT pillars to be obtained by a WACVD process b) False colour image of two selected CNT pillar architectures with cultured cortical neurons (same dimensions as in Figure 3, height about 400 µm). Note: the distance of the virgin CNT pillars in a) is 100 µm whereas in b) we have deliberately chosen for the neuron growth a substrate with a larger distance of about 200 µm between individual CNT pillars to exemplify the strong attraction even over such macroscopic dimensions. c) Close up view of the vicinity of the vertical aligned CNT structure and the neurons.

After 21 days in vitro the neural network is considered mature and the neurons are observed (see SEM, Figure 5). Cells clearly prefer the CNT sidewalls over the tips and only very few cells grow on top of the vertically aligned CNT structures. The majority of cells follow the given pre-structured linear arrangement of the vertical CNTs, indicating that the neurons are strongly attracted towards the sidewall of the CNT pillars. Cells seem to be able to “climb” onto 3D CNT structures having several 100 µm in height. However, they seem to avoid on purpose a colonisation of the tips of these CNT structures. We could verify this behaviour in parallel experiments by modifying size and shape of the CVD grown micro-structured vertically aligned CNT. Neurons are indeed strongly attracted by the micro-shape and especially by the sidewalls of the aligned nanotubes arrangements (see Supporting Information File 1 for details). With the very recently reported ability to tailor the hydrophilicity and hydrophobicity of such 3D aligned CNT structures over a wide range from superhydrophilic to superhydrophobic [29] the directional cell growth on such structures should be possible and would thus allow understanding these observed preferences from a surface chemistry viewpoint in future work.

In conclusion, pristine randomly and vertically aligned CNTs architectures were studied with respect their use as substrates for neuron cell growth. Both CNT architectures are unique hierarchical structures to direct and control neural cell growth. To the best of our knowledge it is reported for the first time that neuron cells prefer sidewalls of high aspect ratio carbon CNT arrangements over the tips of such structures. Neurons grow all the way from the bottom to the top of these CNT architectures. This finding is crucial for the future design of novel scaffolds and guidance systems for cells.

## Supporting Information

File 1 Additional growth studies through SEM.
