# Supplementary material for: Growth and structural discrimination of cortical neurons on randomly oriented and vertically aligned dense carbon nanotube networks
Source: Beilstein J Nanotechnol. 2014 Sep 17;5:1575–9. doi: 10.3762/bjnano.5.169 (PMC4168933; doi:10.3762/bjnano.5.169)
Supplement: File 1 — Additional growth studies through SEM. [file Beilstein_J_Nanotechnol-05-1575-s001.pdf]

Supporting Information

for

Growth and structural discrimination of cortical neurons on randomly oriented and vertically aligned dense carbon nanotube networks

Christoph Nick[1,‡], Sandeep Yadav[2,‡], Ravi Joshi[2], Christiane Thielemann*[1] and Jörg J. Schneider*[2]

Address: [1]University of Applied Sciences Aschaffenburg, Department of Engineering, BioMEMS lab, Würzburger Strasse 45, 64743 Aschaffenburg, Germany and [2]Technische Universität Darmstadt, Fachbereich Chemie, Eduard-Zintl-Institut für Anorganische und Physikalische Chemie, Alarich-Weiss-Str. 12, 64287 Darmstadt Germany

E-mail: Christiane Thielemann* - christiane.thielemann@h-ab.de;

Jörg J. Schneider* - joerg.schneider@ac.chemie.tu-darmstadt.de

* Corresponding author

‡ Equal contributors

Additional growth studies through SEM

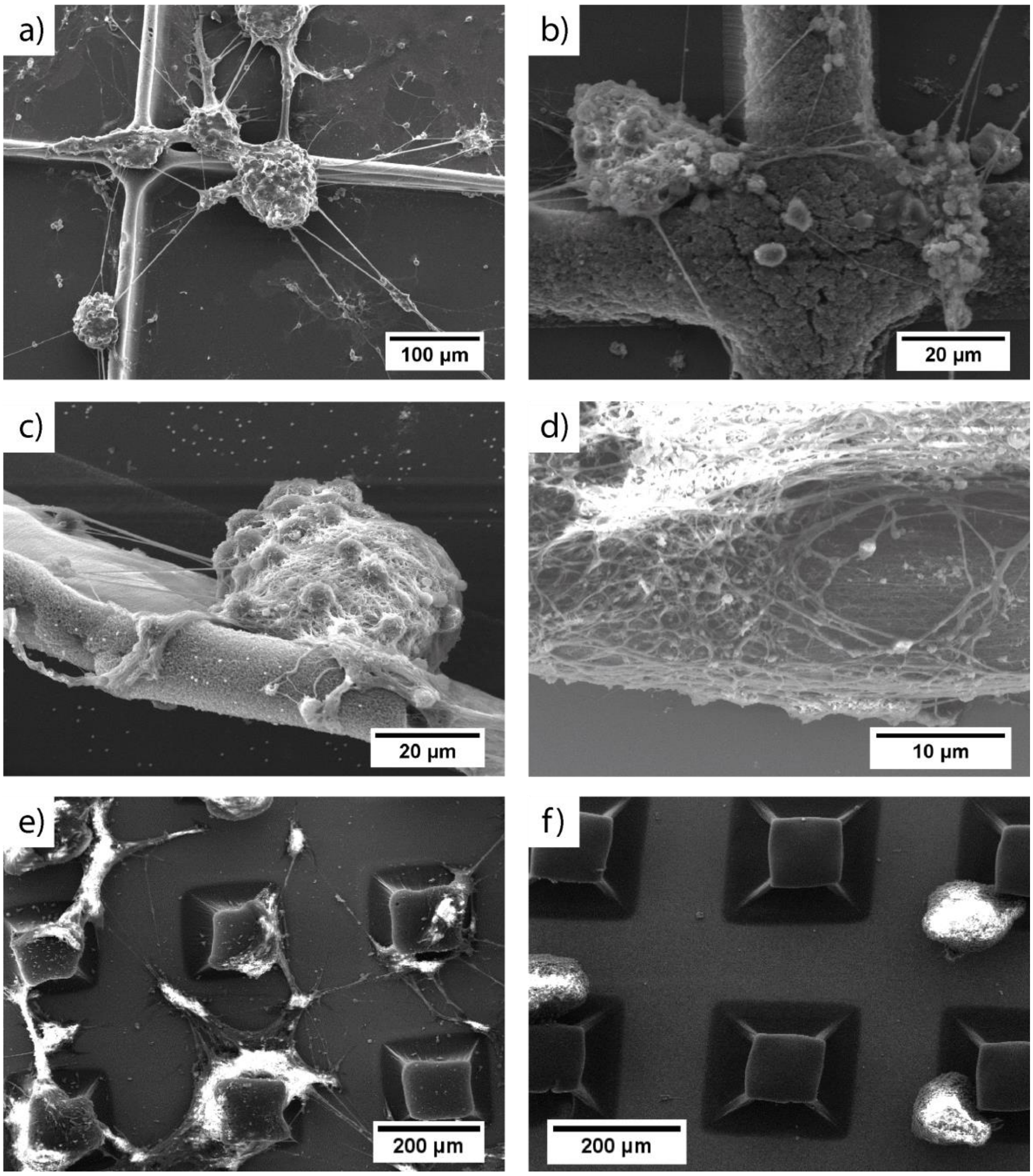

Figure S1: SEM pictures of neuronal cell assembly on different CNT micro architectures. Upper left a): neuronal cell assembly on a CNT cross bar structure. Upper right b): Magnified view of a neuronal cell adhesion on a cross bar CNT structure. Middle left c): Higher magnified view of a neuronal cell cluster showing its preferential adherence on the side architecture of the CNT wall structure. Middle right d): higher magnified view of a dense cell assembly on the CNT upper rim wall structure shown in c). Lower left e): Example emplifying a comparable preferential cell assembly of the neuron cell line R18 (Embryonic Rat Hyothalamus Cell Line R18 (RHypoE-18)) on the CNT side walls as compared to a) or c). Lower right f): Example showing the preferential cell assembly of a neuronal spheroid (from neurons of a chicken embryo) on the CNT side walls of CNT microstructured blocks.
